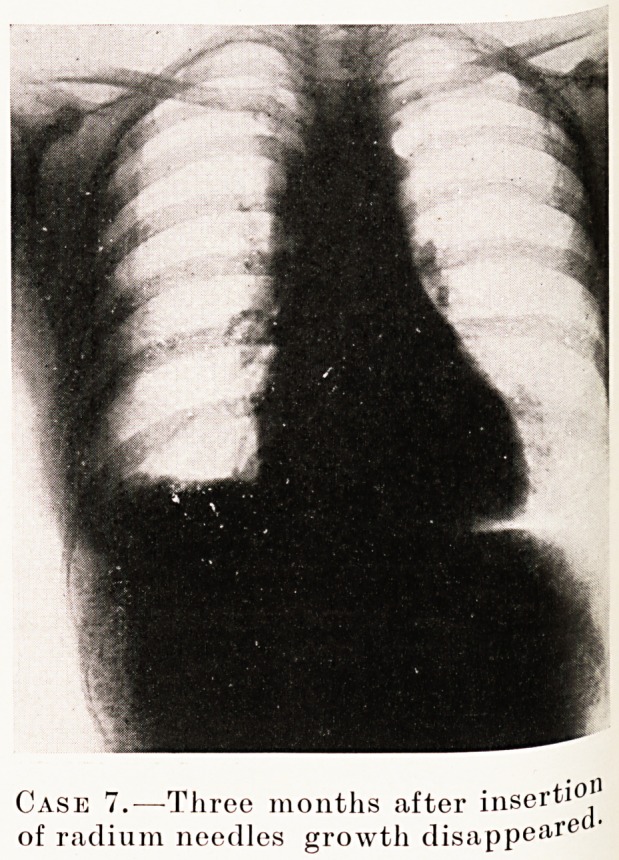# The Treatment of Carcinoma of the Lung

**Published:** 1936

**Authors:** Duncan Wood

**Affiliations:** Surgeon, Bristol General Hospital


### CARCINOMA OF THE BRONCHUS.

### The Treatment of Carcinoma of the Lung.

By DUNCAN WOOD, F.R.C.S.,
*Surgeon, Bristol General Hospital.*

Many obstacles must be faced in treating this condition. I shall not attempt to generalize, but shall describe the results of 14 cases which I have seen since 1930. These cases were sent to me either with a view to surgical treatment or on account of metastatic deposits.

The most radical treatment of carcinoma of the lung is by lobectomy. This, unfortunately, is rarely possible, partly from difficulty in early diagnosis and partly owing to the original site of the lesion. The lesion usually starts in the bronchus, making a radical excision almost impossible. Only 10 per cent. of cases (according to Lilienthal) originate in the parenchyma.

Lobectomy being so rarely possible, treatment with radium has been tried. Surface application of radium was found to be unsatisfactory. It was given in 2 cases: 30 mgm. of radium were applied to the affected side, but had to be removed in a week owing to toxic symptoms (vomiting and tachycardia) apparently due to the breaking down of the neoplasm. Both patients died within a few weeks.

It was therefore decided to try implanting radium directly into the growth, and this has been done in 7 cases. In only one of the series of 14 cases did the growth start in the parenchyma and give hope of treatment by lobectomy :—

**Case 3.**—Male, aged 56. Three months' history of gnawing pain in the right axilla together with loss of weight. No cough, no sputum. The X-ray film shows a well-circumscribed shadow in the upper lobe of the right lung. The Wasserman reaction of the blood was positive, and he was treated with iodides. Two months later he was seen by me and the tumour was exposed by thoracotomy. It was found to be fixed by dense adhesions to the parietal pleura, and could not be removed by lobectomy. 20 mgm. of radium were then implanted into the growth and the needles left in position for seven days. A pleural effusion followed which prevented changes in the tumour being observed. He died seven months later from cachexia.

In the remaining 13 cases the growth started in the bronchus, at the hilum. In 5 of these cases implantation of radium needles by open operation was tried. The anæsthetic used was intra-tracheal ether. The method of approach varied.

*Intercostal thoracotomy.*—For tumours of the lower lobe of the lung, or if lobectomy is under consideration, an intercostal thoracotomy is preferred. This is performed by Shenstone's technique. An intercostal incision is made through the sixth or seventh space, starting from behind the inferior angle of the scapula and ending in front at the costal cartilages. The pleura having been opened, a rib-spreader is introduced. If more room is required the rib above the incision is divided at its vertebral end. The radium needles already threaded are then inserted into the circumference of the tumour, completely surrounding it as far as possible.

With regard to the risk of this operation, in only one case was death hastened. This was not due to the exposure, but to the radium setting up gangrene in an infected lung.

**Case 4.**—Male, aged 58. Admitted December, 1933. History of 12 weeks' pain in the right side. Two weeks blood-stained sputum. Losing weight. Pleural effusion present, blood-stained on aspiration. Operation by intercostal thoracotomy: the inferior lobe of the right lung was found adherent to the diaphragm and a tumour felt at its hilum. Fifteen 1-mgm. needles of radium inserted and left in for ten days. The patient died thirteen days from the date of operation. Post-mortem showed extensive carcinoma of the bronchus with gangrene of the right lower lobe and secondary growths in the glands round the pancreas.

Another case was treated by similar technique:—

**Case 5.**—Male, aged 58. Admitted September, 1935. Illness started with influenza attack three months before, followed by blood-stained expectoration. On admission, dullness over the upper lobe of the left lung. Bronchoscopy and biopsy by Mr. Scarff gave uncertain result. X-ray showed ground glass opacity of the upper area of the left lung. The left upper lobe was exposed by intercostal thoracotomy and was found to be fixed to the chest wall by fine adhesions. These adhesions were separated and 14 mgm. of radium were introduced and left in for seven days. X-ray taken some weeks later showed complete opacity of left side of chest. The hæmoptysis continued. He died five months later from cachexia.

*Anterior mediastinotomy.*—Hoping to improve on these results, another method of approach was tried.

When the tumour is situated at the hilum of the lung a more direct access is obtained by anterior mediastinotomy. This, of course, means dividing the sternum. Bryant's trap-door incision was tried; this gives rather a limited exposure and the possibility of opening both pleural cavities.

Dunhill's modification of Milton's vertical incision through the sternum is better. Having divided the sternum, it is necessary to open the pleural cavity on the side of the tumour. The radium needles are then introduced, surrounding the tumour as completely as possible. Owing to the resulting pneumothorax, removal of the needles has not been found difficult. This is in marked contrast to radium in the peritoneal cavity, where adhesions rapidly form. Irritation of the pleura with resulting pleural effusion is much less marked with mediastinotomy than by intercostal thoracotomy. Three cases have been treated by this technique.

**Case 6.**—Aged 39. Three months ago had a cough for a month. Sent up to hospital for gastric symptoms. Two months' vomiting, a half to one hour after meals, relieved by vomiting. Lost 1½ stone in six months. X-ray shows ground glass opacity of left upper lobe of lung. Bronchoscopy and biopsy by Mr. Scarff; epithelioma. *Anterior mediastinotomy.*—10 mgm. radium inserted and left in for seven days. Case too recent to report result.

**Case 7.**—Female, aged 38. History of swelling in the right arm, noticed in May, 1930, ten weeks before admission ; three weeks' dyspnœa and stridor ; sputum thick and lumpy. On admission acutely ill with dyspnœa, inability to swallow and difficulty in expectoration. X-ray of chest showed shadow bulging to the right of the sternum at its upper level. Bronchoscopy by Mr. Scarff showed a projection pushing in the wall of the right bronchus. Four days later the dyspnœa became so urgent that anterior mediastinotomy was performed. The right pleural cavity was opened and a vascular fixed tumour was found bulging from the mediastinum. Ten 1 mgm. needles of radium were inserted and left in for ten days. The acute symptoms were relieved by the mediastinotomy. An X-ray film taken three months later shows the tumour shadow to have nearly disappeared. Her present symptoms, that is five years later, are shortness of breath on exertion and cough at night. There is a dilated vein on the right side of chest. The right arm is increased 1½ inches in circumference, due to œdema. An X-ray with lipiodol shows that the upper lobe of the bronchus does not fill.

As no biopsy was done there is no actual proof that this tumour was a carcinoma. Clinically, at the time, this diagnosis was suggested. There is no doubt that the mediastinotomy relieved the acute

### PLATE XV

Case 3.—Parenchymatous growth of right upper lobe.

Case 3.—Radium needles in situ. Pleural effusion following intercostal thoracotomy.

Case 6.—Incision used for anterior mediastinotomy.

Left bronchial carcinoma.

Case 6.—Radium needles in situ; introduced by anterior mediastinotomy.

Note.—The position of the needles is shown by white marks: in the actual skiagram they are of course *dark* shadows.

PLATE XVI

CASE 7. — Showing growth protruding from upper right mediastinum.

CASE 7.—Three months after insertion of radium needles growth disappeared.

symptoms, and that the radium caused the tumour to disappear.

**Case 8.**—Male, aged 61. Admitted October, 1935, with three months' cough and hæmoptysis. Losing weight. Two months' pain in the chest. Sputum showed cells very suspicious of malignancy. X-ray showed shadow with ill-defined outline growing from the left hilum. Bronchoscopy by Mr. Scarff, biopsy finding negative. Treated by anterior mediastinotomy. Five 1 mgm. needles of radium inserted and left in for ten days. The treatment has stopped his hæmoptysis. Mr. Scarff is introducing more radium by bronchoscopy. In the future we hope to treat more cases by this double method of approach.

### CASES UNSUITABLE FOR RADIUM TREATMENT.

Abscess formation may dominate the clinical picture. The diagnosis of malignancy may then become extremely difficult. The abscess may be either in the lung or in the pleural cavity. Surgical treatment is confined to drainage of the abscess. Two cases occurred in this series :

**Case 9.**—Male, aged 54. History of two weeks' dyspnœa. Admitted with afebrile empyema. Needled, pus found, culture pneumococcal. Resection of rib with drainage. Rapidly developed cerebral and mental symptoms with hemiplegia. Post-mortem revealed primary carcinoma of bronchus with secondaries in brain, liver and suprarenals.

**Case 10.**—Male, aged 46. Six months' history of tightness in the throat and cough and irregular temperature. X-ray showed large, irregular shadow in upper lobe of left lung. Needling failed to find pus. Operation, local rib resection with drainage of lung abscess. Culture, pus staphylococcal. Died of cachexia.

Of the remaining 4 cases two patients were too ill when first seen : one of these died from acute hæmoptysis, and the other died in eighteen days with secondary growths in the liver. In this case the history was of six weeks' abdominal pain, hæmoptysis starting only six days before death. In the other 2 cases the symptoms were due to metastatic growths.

**Case 13.**—Male, aged 54. Symptoms, pain in right thigh. Spontaneous fracture whilst being examined. X-ray showed rarefaction of right femur, with absorption of cortex, but no expansion. Left humerus showed suspicious rarefaction. Four days later this also fractured. He died in eight weeks. Post-mortem showing carcinoma of bronchus.

**Case 14.**—Male, aged 70. For six months felt lump in chest wall; losing weight; chronic cough for eight years. Two years' sputum, blood-stained at times. On admission, hard irregular tumour attached to the right pectoral muscle, not fixed to chest wall. X-ray shows shadow growing from right hilum. Biopsy of tumour, secondary epithelioma. Patient died two months later from cachexia.

### SUMMARY.

1. Lobectomy is the best treatment for carcinoma of the lung, but it can only be done at a relatively early stage, and if the lesion is in the parenchyma.

2. In carcinoma of the hilum, if implantation of radium into the growth is under consideration, then access by anterior mediastinotomy gives the most direct approach and causes less irritation to the pleura.

3. In some of the cases the diagnosis of malignancy can only be made by exploration. If not malignant the tumour can then be enucleated. Non-malignant tumours in this site, if not removed, are ultimately fatal.

## Figures and Tables

**Case 3. Case 3. Case 6. Case 6. f1:**
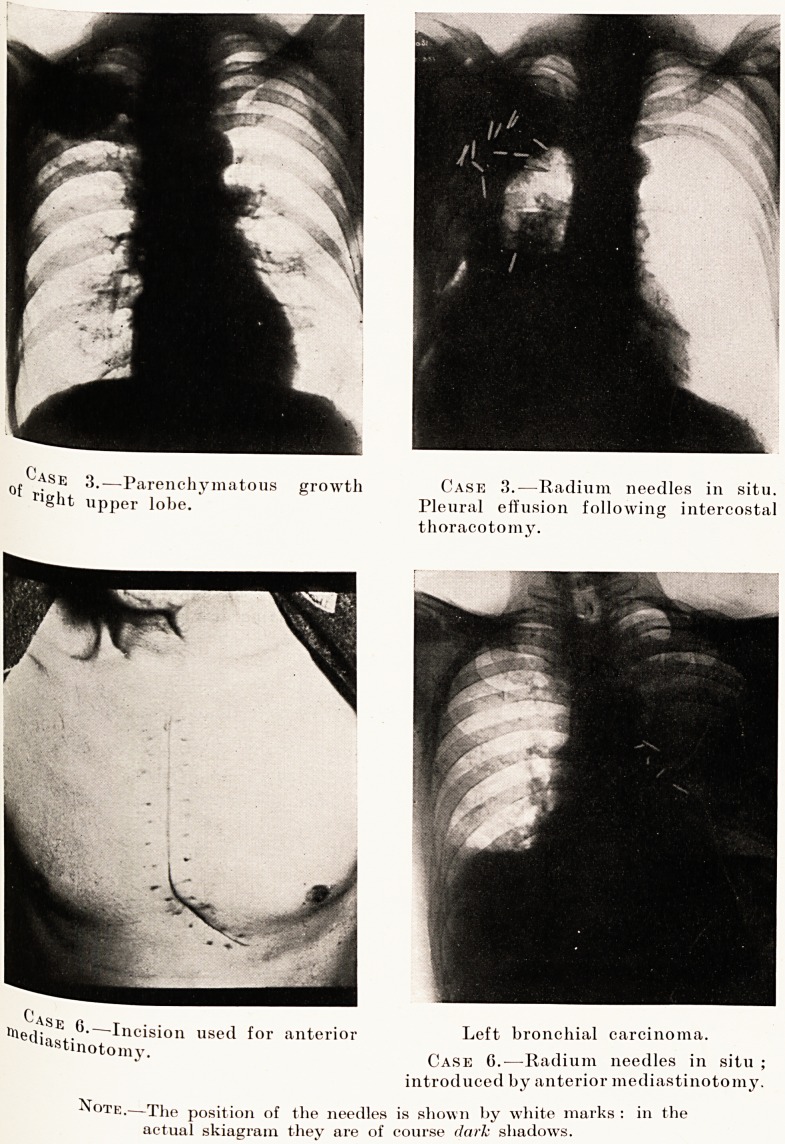


**Case 7. f2:**
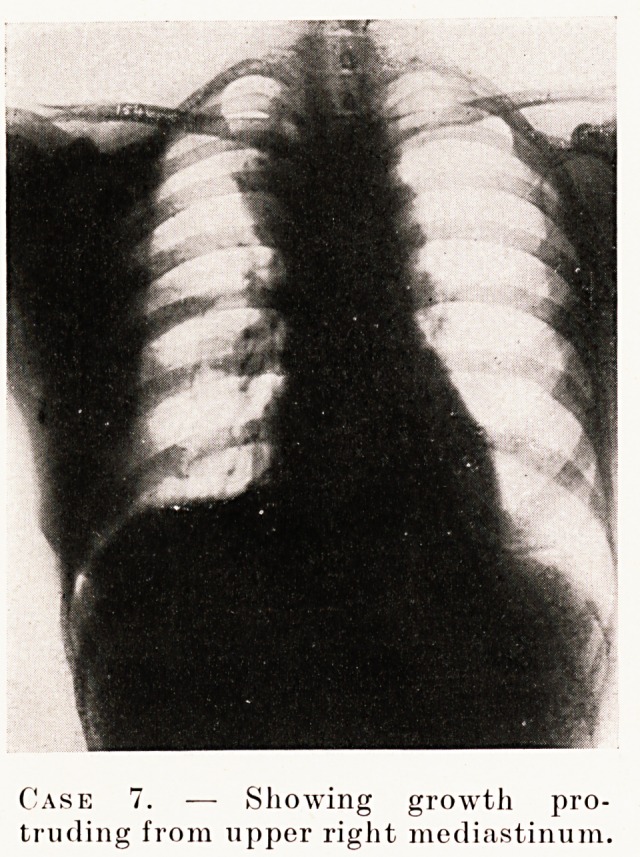


**Case 7. f3:**